# Glycoside rich fraction from *Spondias pinnata* bark ameliorate iron overload induced oxidative stress and hepatic damage in Swiss albino mice

**DOI:** 10.1186/s12906-016-1244-4

**Published:** 2016-07-29

**Authors:** Dipankar Chaudhuri, Nikhil Baban Ghate, Sourav Panja, Tapasree Basu, Anil Khushalrao Shendge, Nripendranath Mandal

**Affiliations:** Division of Molecular Medicine, Bose Institute, P 1/12, C. I. T. Road, Scheme – VIIM, Kolkata, 700054 West Bengal India

**Keywords:** Antioxidant, Iron chelation, Histopathology, Phytochemicals, Hepatic damage, Serum enzymes

## Abstract

**Background:**

Iron in the overloaded condition in liver promotes the overproduction of free radicals that lead to oxidative stress and ultimately hepatic damage. The present study was designed to evaluate the ameliorating potential from iron overloaded hepatotoxicity by the glycosidic fraction from *Spondious pinnata* bark (SPW1) along with its antioxidant property.

**Methods:**

The fraction was tested for its in vitro antioxidant, free radical scavenging property and iron chelation potential via standard biochemical assays. Iron overload condition was generated by the intraperitoneal administration of iron dextran in mice. The levels of serum enzymes, antioxidant enzymes in liver, markers of hepatic damage, liver iron, and ferritin content were measured in response to the oral treatment of SPW1. Histopathology of the liver sections was performed for visual confirmation of the amelioration potential of SPW1.

**Results:**

The fraction exhibited excellent in vitro antioxidant as well as free radical scavenging potential against both reactive oxygen species and reactive nitrogen species. Administration of SPW1 significantly normalized the disturbed levels of antioxidant enzymes, liver iron, lipid peroxidation, liver fibrosis, serum enzyme and ferritin better than standard desirox which were also supported by the morphological study of the liver sections. Phytochemical analysis as well as HPLC study, confirmed that the fraction mainly consisted of glycosidic phenolics and flavonoids that attributed to its biological activities.

**Conclusions:**

The above results suggested that beneficial effects of SPW1 on iron overload induced hepatotoxicity that can be considered as a possible candidate against iron overload diseases.

## Background

Liver, the largest glandular organ in the body, plays a dynamic role in metabolism, biotransformation of proteins, carbohydrates, lipids and also detoxification of different endogenous and exogenous xenobiotics and drugs. Hence, the liver is liable to injury due to the chronic exposure to environmental toxicants, drugs and other xenobiotics [1]. Nowadays, iron overload mediated disorders like liver fibrosis, cirrhosis, inflammation, diabetes, impaired cardiac function, arthritis and even cancer [2], are common throughout the world occurring mostly in major race/ethnic groups [3]. Moreover, iron overloaded toxicity caused a major problem in thalassemic patients than other type of liver toxicity. Although, iron, one of the most important redox metals, is essential for different cellular processes as there is a close relationship between iron essentiality and iron toxicity. Excess iron causes a serious damage to the liver that is the main storage site of iron in our body, by forming hydroxyl radical mediated oxidative stress. So, protection against metal toxicity by chelating metal ions or by trapping free radicals (antioxidant) is an effective life-saving strategy for nearly all the iron overload-induced diseases mentioned above [4]. In addition to endogenous antioxidant systems, consumption of natural supplements that is rich in natural antioxidants also exhibits increased resistance to oxidative stress by altering the redox environment and is associated with a lower risk of many oxidative stress-related diseases.

Different phytochemicals mainly natural water soluble phenolics and flavonoids are used to treat iron-induced liver toxicity as they can efficiently scavenge most of the free radicals through their relevant iron chelating capabilities. From the previous publications, it was observed that 70 % methanol extract of *Spondias pinnata* (Linn. f.) Kurz (Fam. Anacardiaceae) possessed both in vitro & in vivo antioxidant and iron-chelating potential, which was supported by the presence of significant amounts of phenolics and flavonoids [5, 6]. These previous studies prompted us to separate the water soluble glycosidic compounds from *S. pinnata* bark and evaluate their ameliorating effect on iron overload-induced hepatotoxicity and hepatic fibrosis in mice.

## Methods

### Reagents

2,2′-azinobis-(3-ethylbenzothiazoline-6-sulfonic acid) (ABTS) was purchased from Roche diagnostics, Germany and 6-hydroxy-2,5,7,8-tetramethylchroman-2-carboxylic acid (Trolox) was obtained from Fluka, Switzerland. Bathophenanthrolinesulfonate disodium salt, 5,5′-dithiobis-2-nitrobenzoic acid (DTNB) and N-(1-Naphthyl) ethylenediaminedihydrochloride (NED) were procured from Sisco Research Laboratories Pvt. Ltd, India. 1-chloro-2,4-dinitrobenzene (CDNB), Dimethyl-4-aminobenzaldehyde, and N,N- dimethyl-4-nitrosoaniline ammonium iron (II) sulfate hexahydrate ((NH_4_)_2_Fe(SO_4_)_2,_ 6H_2_O) were obtained from Merck, Mumbai, India. Guanidine hydrochloride and Iron-dextran was purchased from Sigma-Aldrich, USA. Desirox (Deferasirox) was obtained from Cipla Ltd., India. All of the other used reagents were of molecular biology grade and were obtained from reputable suppliers.

### Test animals

In vivo experiments were performed abiding by the guidelines of the Committee for the Purpose of Control and Supervision of Experiments on Animals (CPCSEA), Ministry of Environment and Forest, Govt. of India with due approval from the Institutional Animal Ethics Committee, Bose Institute (Registration. No. 95/1999/CPCSEA). Male Swiss albino mice (20 ± 2 g) were obtained from the Chittaranjan National Cancer Institute (CNCI), Kolkata, India and were acclimated under a constant 12 h light / dark cycle at 22 ± 2 °C. The animals were fed general laboratory diet and water ad libitum. Experimental animals were taken care every six hour during the treatment period, and it was observed that there was no unwanted animal death. All surgeries were done using ethyl ether as an anesthetic, taking utmost care to reduce suffering.

### Plant material

*S. pinnata* bark was collected from the villages of Bankura district, West Bengal, India and the plant was authenticated by Dr. Jayaram Hazra, Director, Central Research Institute of Ayurveda (CRIA), Kolkata, India. The herbarium was submitted at CRIA, Kolkata with an accession no of CRHS 111/08.

### Fractionation of crude extract

The powdered *S. pinnata* stem bark was extracted with 70 % methanol and water [5]. The lyophilized extract was re-extracted successively with hexane, chloroform, ethyl acetate and water. The water fraction again fractionated by acetylation (2 ml of pyridine and 2 ml of acetic anhydride was stirred with 500 mg of water fraction at 40 °C for 6 h) followed by silica gel column chromatography purification (major spot) and deacetylation (150 mg of sodium methoxide was stirred with 500 mg of acetylated product dissolved in 50 % methanol in dichloromethane at room temperature for 6 h) to get the fraction namely SPW1.

### In vitro study

#### Antioxidant potentials

Antioxidant capacities of SPW1 was evaluated by ABTS^•+^ radical cation decolorization assay, DPPH (2,2-diphenyl-1-picrylhydrazyl) scavenging assay, and Fe^3+^-reducing power assay according to a standard method [5]. The scavenging percentage was quantified using test and control experiment values.

#### Free radical scavenging activity

In vitro reactive oxygen species (ROS) scavenging properties were determined by superoxide, hydroxyl, hypochlorous and singlet oxygen radical scavenging assays, and reactive nitrogen species (RNS) scavenging activity was determined by nitric oxide and peroxynitrite radical scavenging assays following standard procedures [5].

#### In vitro iron chelation and ferritin iron release

The Fe^2+^ chelating ability was determined as described earlier, and the result was expressed as inhibition percentage [5]. Iron reduction and release were determined using ferrozine, a spectrophotometric reagent for iron, as previously described [7]. Briefly, the reaction was initiated by adding different concentrations (100–500 μg/ml) of test compounds in 50 mM phosphate buffer (pH 7.0) containing 200 μg ferritins and 500 μM ferrozine, and the absorbance change was measured for 20 min at 560 nm.

### In vivo study

#### Experimental design

In total, six groups of mice were randomly prepared to consist of six mice per group. Among them, one group received normal saline only, which was labeled as a blank (B); however, other groups were intoxicated with five doses of 100 mg/kg b.w. iron-dextran (one dose every alternative day) by intraperitoneal injection (ip). Of the groups, one iron-dextran group (C) was treated orally with the only saline, and other groups were treated with 50 mg/kg b.w SPW1 (S50), 100 mg/kg b.w. SPW1 (S100), 200 mg/kg b.w. SPW1 (S200), test samples and 20 mg/kg b.w. desirox (D) for 21 consecutive days starting on the day following the first iron-dextran injection. The doses of SPW1 were selected on the basis of the obtained LD_50_ value (1309 mg/kg b.w.). All experimental animals were sacrificed on the 22nd day under mild anesthesia (ethyl ether), and the cardiac puncture was performed to collect blood and serum was separated and stored at −80 °C. After collecting the blood, the liver was quickly excised, cleaned thoroughly with cold phosphate buffer saline (PBS) to remove the remaining blood and cut into three sections. The major liver portion was dissected and homogenized using 10 volumes of 0.1 M phosphate buffer (pH 7.4) supplemented with 0.15 M NaCl and 5 mM EDTA and centrifuged for 30 min at 8000 g in the cold. The clear homogenate (supernatant) was collected and the protein concentration was quantified by the Folin-Lowry method, where BSA was used as a standard; the remaining supernatant was then stored at −80 °C. The second liver fragment was treated with a mixture of nitric acid and sulfuric acid (1:1) to analyze the iron content. The last portion was processed for histopathological examinations. This procedures were performed according to pervious method [6].

#### Serum markers

Aspartate aminotransferase (ASAT), alanine aminotransferase (ALAT), and bilirubin levels in serum were evaluated using commonly available kits from Merck, India. Similarly, alkaline phosphatase (ALP) levels were measured by a kit from Sentinel Diagnostics, Italy.

#### Antioxidant enzymes

Superoxide dismutase (SOD), catalase (CAT), glutathione-S-transferase (GST), and reduced glutathione (GSH) levels were measured using previously described methods [6].

#### Evaluation of liver damage and fibrosis

Thiobarbituric acid reactive substances (TBARS) quantities were measured to evaluate the levels of lipid peroxidation. Protein oxidation levels were resolved by estimating protein carbonyl contents. Collagen content, an important marker of liver fibrosis in each sample was determined by multiplying 7.69 to overall hydroxyproline content. These values were measured using previously described methods [6].

#### Serum ferritin and liver iron levels

Manufacturer’s instructions were followed to quantify serum ferritin levels using an ELISA kit from Monobind Inc., USA. Liver iron content was quantified using a previously reported method [6]. Briefly, samples were mixed with bathophenanthroline sulfonate and incubated at 37 °C for 30 min, and absorbance was recorded using a spectrophotometer at 535 nm.

#### Histopathological studies

Excised liver samples were cleaned with saline and fixed for two days in 10 % buffered neutral formalin. Sections (5 μm thick) were paraffin-embedded and stained with hematoxylin and eosin (morphological examination), Perls’ Prussian blue dye (iron content) and Masson’s trichrome stain (liver fibrosis). Stained sections were checked microscopically for histopathological changes.

#### Phytochemical analysis

The presence of phytochemicals was evaluated following different standard methods [8, 9]. HPLC analysis was performed according to the previous method to identify the bioactive phytochemicals [10] present in SPW1.

#### Statistical analysis

All of the data were reported as the mean ± SD of six measurements. Statistical analysis was performed using KyPlot version 2.0 beta 15 (32 bit) and Microsoft Excel 2010. The relationships between the groups were evaluated using a paired *t*-test, and a *p* value of <0.05 was considered to be statistically significant.

## Results

### Fractionation of extract

The polarity based fractionation by different solvents revealed that the water fraction yielded more than 90 % of the total extract, indicating the fact that the activity of the crude extract was mainly due to water soluble polar compounds. Further to regroup the probable glycosidic compounds in water fraction, it was acetylated to convert the polar acid groups into non-polar acetyl group. This process would facilitate the separation of the similar group of compounds through silica gel chromatography. The major spot, among the other several minor spots, was isolated by silica gel column chromatography and further deacetylated to remove the acetyl groups to revert the native free carboxylic acid groups of the compounds in that fraction.

### In vitro study

#### Antioxidant potentials

**Fig. 1:**
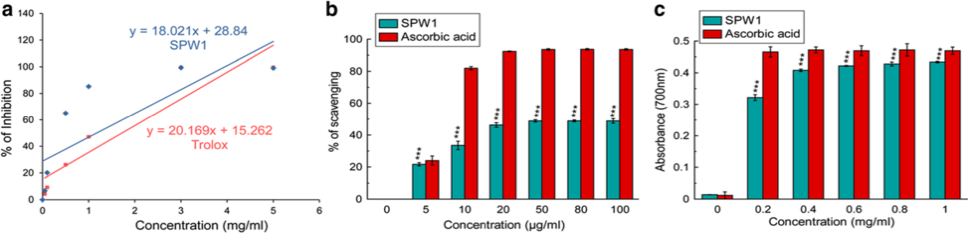
Antioxidant potentials of SPW1. **a** Total antioxidant assay, **b** DPPH radical scavenging assay, **c** Reducing power assay. The results represent the mean ± S.D. (*n* = 6). ***p < 0.001 vs. control

The overall picture of the antioxidative ability of SPW1 was evaluated by ABTS^•+^ radical cation scavenging, DPPH radical scavenging and reducing power capacity assays. It was observed that SPW1 possessed excellent total antioxidant capacity that is almost similar to the standard Trolox (Fig. 1a). Similarly the fraction also strongly scavenge DPPH radical as well as reduce the Fe^3+^ to Fe^2+^ by donating an electron which also support the antioxidative capacity of SPW1 (Fig. 1b, c respectively). The TEAC and the IC_50_ values listed in Table 1.

**Table 1:** IC_50_ values of the SPW1 and standard compounds for different antioxidant, free radical and metal chelation assays

Name of assay	SPW1	Standards	Values of standard compounds
DPPH	50.76 ± 1.49	Ascorbic acid	5.29 ± 0.28
Superoxide anion (O_2_•^−^) scavenging	8.95 ± 0.29	Quercetin	42.06 ± 1.35
Hydroxyl radical (OH•) scavenging	7244.91 ± 905.97	Mannitol	571.45 ± 20.12
Hypochlorous acid (HOCl) scavenging	66.05 ± 1.92	Ascorbic acid	235.96 ± 5.75
Singlet oxygen (^1^O_2_) scavenging	202.81 ± 7.95	Lipoic acid	46.15 ± 1.16
Nitric oxide radical (NO) scavenging	121.55 ± 5.01	Curcumin	90.82 ± 4.75
Peroxynitrite (ONOO-) scavenging	403.17 ± 8.01	Gallic acid	876.24 ± 56.96
Iron chelating activity	59.98 ± 0.67	EDTA	1.27 ± 0.05

All the values are expressed in μg/ml. Data expressed as mean ± S.D (*n* = 6). EDTA represents Ethylenediamine tetraacetic acid

#### Free radical scavenging activity

**Fig. 2:**
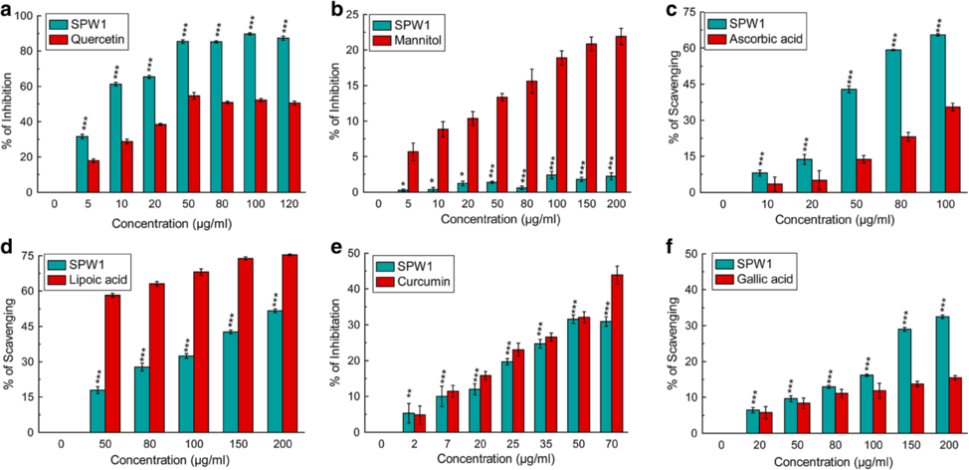
ROS and RNS scavenging capabilities of SPW1. **a** Superoxide radical inhibition, **b** Hydroxyl radical inhibition, **c** Hypochlorous radical scavenging, **d** Singlet oxygen radical scavenging, **e** Nitric oxide inhibition, **f** Peroxynitrite radical scavenging. The results represent the mean ± S.D. (*n* = 6). *p < 0.05, **p < 0.01 and ***p < 0.001 vs. control

The free radicals scavenging activity of the fraction SPW1 against different ROS & RNS was depicted in the Fig. 2, and the IC_50_ values were listed in Table 1. From the Fig. 2 it was clear that the fraction scavenged/inhibited different ROS, and RNS especially superoxide, hypochlorous acid, peroxynitrite radicals since the activity of the fraction was better than the standards (Fig. 2a, c, f respectively). On the other hand, SPW1 exhibited moderate activity against singlet oxygen and nitric oxide radicals (Fig. 2d, e respectively) but failed to scavenge hydroxyl radical (Fig. 2b).

#### In vitro iron chelation and ferritin iron release

**Fig. 3:**
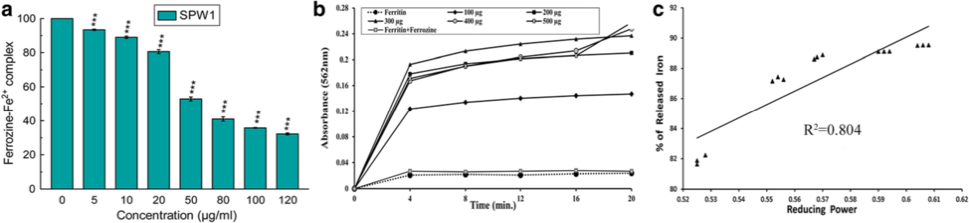
In vitro iron chelation and reductive release of ferritin iron (Fe^3+^). **a** Iron chelation activity **b** Iron release from ferritin **c** Correlation between iron released from ferritin with reducing power

Iron (Fe^2+^) chelation capability of SPW1 was determined by hindering the development of violet colored Fe^2+^-ferrozine (Fig. 3a). At the highest tested concentration, SPW1 disrupted the complex color formation up to 68 %. From the Fig. 3b it was observed that SPW1 effectively released iron (Fe^3+^) from ferritin dose-dependently and also displayed significant positive correlation (R^2^ = 0.8041) between the reducing power and (%) the iron released from ferritin (Fig. 3c).

### In vivo study

#### Liver iron and serum ferritin levels

**Fig. 4:**
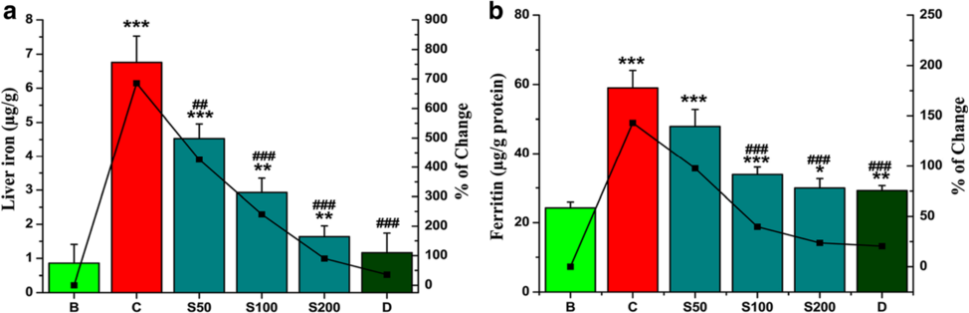
Removal of overloaded iron from mice liver and blood serum. **a** Iron load in liver cell, **b** Level of ferritin in serum. Mouse groups (B, C, S50, S100, S200, D) were treated as described in the ‘Experimental design’ section. Values are expressed as the mean ± SD of six mice. *p < 0.05, **p ≤ 0.01, ***p ≤ 0.001 compared with the blank and ^#^ *p* < 0.05, ^##^ *p* < 0.01, ^###^p ≤ 0.001 compared with the control

The iron overload condition was measured directly by the amount of iron in the liver and indirectly by ferritin content in serum. Compared to the normal mice the iron content (686 %) and serum ferritin (143 %) was elevated due to iron overload but on treatment with SPW1 reverted the iron content as well as ferritin content almost to the levels of normal mice (Fig. 4a, b respectively) and the EC_50_ values were listed in Table 2.

**Table 2:** EC_50_ values of SPW1 in deferent assays

Name of the assays	EC_50_ of SPW1 (mg/kg.b.w.)
Serum ferritin and liver iron levels	
Serum Ferritin	177.491 ± 8.80
Liver Iron	81.15 ± 11.57
Serum Markers	
ALAT	73.77 ± 4.19
ASAT	140.01 ± 16.99
ALP	159.32 ± 8.79
Bilirubin	263.18 ± 15.65
Antioxidant enzymes	
SOD	27.99 ± 11.90
Catalase	84.29 ± 11.27
GST	90.84 ± 10.92
GSH	389.19 ± 25.68
Liver damage parameter	
Lipid peroxidation content	223.10 ± 8.65
Protein carbonyl content	171.04 ± 11.40
Collagen content	205.59 ± 7.26

#### Serum markers

Intoxication with iron in mice liver elevated the serum ALAT, ASAT, ALP and bilirubin that denoted hepatic injury. As per Table 3, the levels of serum markers gradually dropped dose-dependently at the individual level on treatment with SPW1 and the EC_50_ values were listed in Table 2.

**Table 3:** The changes in ALAT, ASAT, ALP and Bilirubin levels after oral therapy with SPW1

Treatment	ALAT		ASAT		ALP		Bilirubin	
	Unit/L	% change	Unit/L	% change	Unit/L	% change	mg/dl	% change
B	14.42 ± 1.91		62.46 ± 6.32		84.62 ± 3.48		1.58 ± 0.10	
C	64.60 ± 0.73 ^X3^	347.88	234.91 ± 8.30 ^X3^	276.13	252.63 ± 4.92 ^X3^	198.56	3.15 ± 0.18 ^X3^	99.67
S50	36.19 ± 1.16 ^X3Y3^	150.91	170.49 ± 12.54 ^X3Y3^	172.97	181.71 ± 6.18 ^X3Y3^	114.74	2.42 ± 0.25 ^X2Y2^	53.43
S100	27.47 ± 1.55 ^X3Y3^	90.45	137.29 ± 17.82 ^X3Y3^	119.82	149.48 ± 7.02 ^X3Y3^	76.66	2.18 ± 0.12 ^X3Y3^	38.41
S200	20.11 ± 0.16 ^X2Y3^	39.41	97.62 ± 8.16 ^X3Y3^	56.31	125.08 ± 5.93 ^X3Y3^	47.81	1.99 ± 0.13 ^X3Y3^	26.22
D	19.85 ± 0.79 ^X3Y3^	37.59	88.06 ± 10.37 ^X2Y3^	40.99	114.97 ± 6.13 ^X3Y3^	35.87	1.72 ± 0.14 ^X1Y3^	8.92

Mouse groups (B; C; S50, S100, S200, D) were treated as described in ‘Experimental design’ section. Values are mean ± SD (*n* = 6). X1: *p* < 0.05, *X*2: *p* < 0.01 and X3: *p* < 0.001 significant difference from B group. Y1: *p* < 0.05, Y2: *p* < 0.01 and Y3: *p* < 0.001 significant difference from C group

#### Antioxidant enzymes

**Fig. 5:**
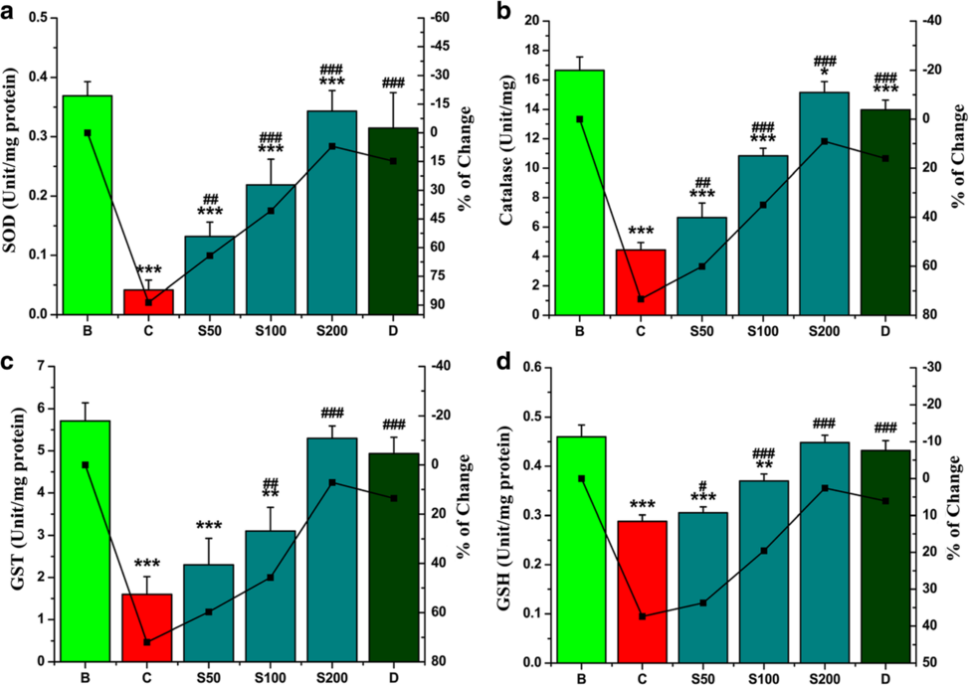
Restoration of liver antioxidant enzyme levels after treatment with SPW1. **a** SOD, **b** Catalase, **c** GST, **d** GSH. Mouse groups (B, C, S50, S100, S200, D) were treated as described in the ‘Experimental design’ section. Values are expressed as the mean ± SD (*n* = 6). *p < 0.05, **p ≤ 0.01, ***p ≤ 0.001 compared with the blank and ^#^ *p* < 0.05, ^##^ *p* ≤ 0.01, ^###^p ≤ 0.001 compared with the control

Iron in overloaded condition induced oxidative stress and reduced the levels of antioxidant enzymes. After the treatment with SPW1, the dropped levels of SOD (89 %), Catalase (73 %), GST (72 %), GSH (37 %) were dose-dependently restored nearly to the normal condition (Fig. 5a, b, c, d respectively) and the EC_50_ values were listed in Table 2. The effect of restoring the antioxidant enzymes level by SPW1 is better than desirox.

#### Evaluation of liver damage and fibrosis

**Fig. 6:**
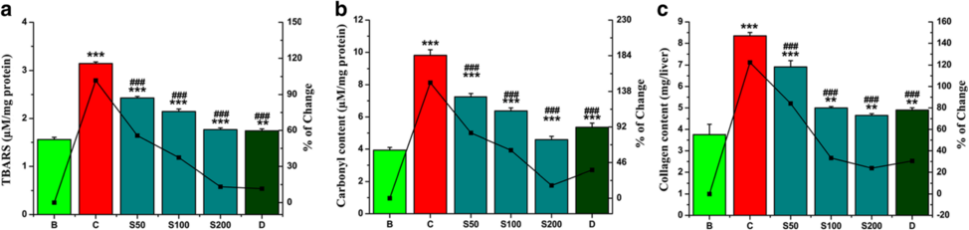
SPW1 treatment gradually reduced the elevated levels of liver damage parameters. **a** Lipid peroxidation levels, **b** Protein carbonyl content, **c** Collagen content. Mouse groups (B, C, S50, S100, S200, D) were treated as described in ‘Experimental design’ section. Values are expressed as the mean ± SD (*n* = 6). **p ≤ 0.01, ***p ≤ 0.001 compared with the blank and ^###^ *p* ≤ 0.001 compared with the control

The effect of iron overload on liver damage was measured by the level of lipid peroxidation, protein carbonyl and collagen content in the liver homogenate. The increased levels of these liver damage and fibrosis markers were reduced after oral administration of SPW1 (Fig. 6a, b, c respectively) and the EC_50_ values were listed in Table 2.

#### Histopathological studies

**Fig. 7:**
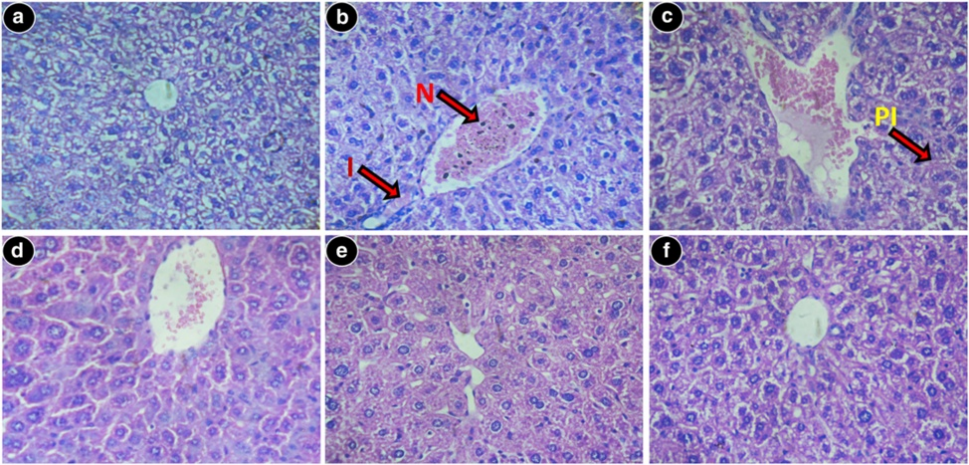
Morphological evaluation of liver sections, stained with hematoxylin and eosin at × 400. **a** Control mice liver with normal cyto-structure. **b** Iron-overloaded liver section showed disrupted cell membrane, inflammation (*I*), and necrosis (*N*). **c** Liver section from the S50 group showed improved histology with portal inflammation (*PI*). **d** Liver section from the S100 group. **e** Liver section from the S200 mouse group. **f** Cellular morphology is almost similar to Desirox-treated liver sections

**Fig. 8:**
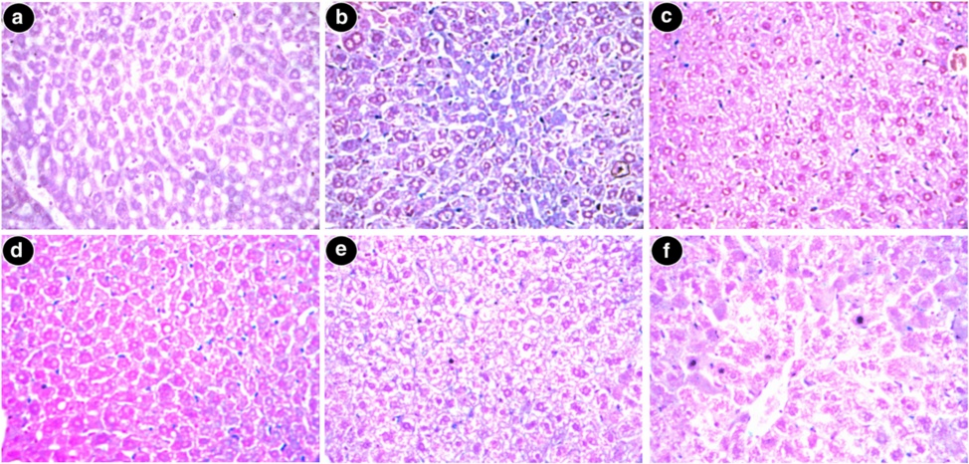
Visual confirmation of iron removal potential of SPW1 by liver sections stained with Perls’ Prussian blue at × 400. **a** Control mice with minimal hemosiderin deposition patches (very low). **b** Excess blue patches denote iron-overloaded condition in mice. **c** Liver section from S50 mouse group. **d** Liver section from S100 mouse group **e** Liver section from S200 mouse group. All three groups (C, D, E) showed gradual reduction of the blue patches. **f** Desirox-treated liver section with lesser blue patches

**Fig. 9:**
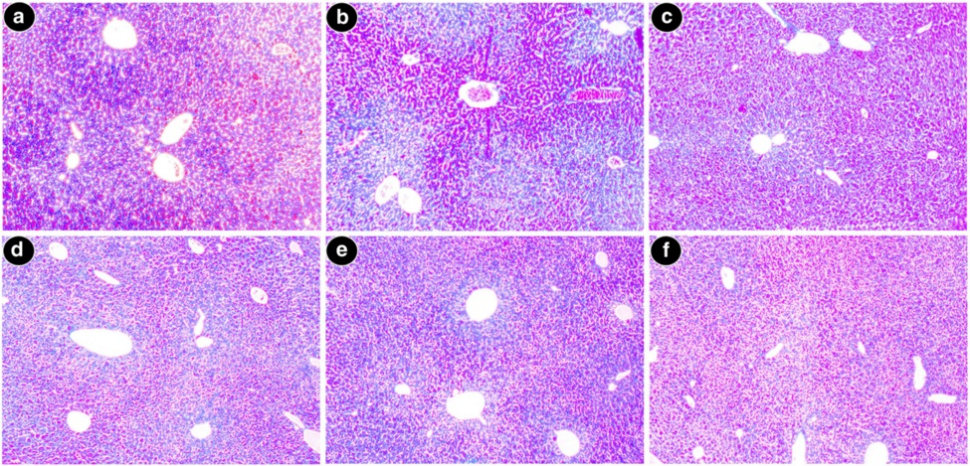
Reduction of liver fibrosis observed by staining the liver sections with Masson’s Trichrome × 100. **a** Control mice showed no sign of fibrosis. **b** Iron-intoxicated mice displayed elongated fibrous septa and collagen accumulation (blue). **c** Liver section from S50 with lesser blue patches, **d** Liver section from S100 group **e** S200 demonstrates a nearly negligible collagen accumulation and healthy liver. **f** Desirox-treated liver section

Liver sections stained with hematoxylin and eosin from normal mice demonstrated normal cell morphology with prominent nuclei in the well-preserved cytoplasm and prominent central vein without cellular infiltration (Fig. 7a). Whereas iron dextran overloaded mice demonstrated various degrees of pathological changes including ballooning degeneration, inflammation, loss of cellular boundaries and hepatocellular necrosis (Fig. 7b). In contrast, SPW1 treated mice groups displayed attenuation of pathogenicity and gradual reversal to normal cytoarchitecture, thus restoring the normal condition (Fig. 7c, d, e respectively). Figure 7f represents liver sections of the desirox-treated group with improved histology, which is similar to the highest dose of SPW1. Another detrimental effect of excess iron in the liver is deposition of iron in the form of crystalline ferritins and amorphous hemosiderin. Iron released from denatured ferritin, ferric oxide (unused iron) as well as broken hemoglobin formed a complex to store the iron known as hemosiderin. Perls’ Prussian blue is commonly used to detect its deposition in liver tissue as blue patches. The liver sections from untreated iron overloaded mice demonstrated increased hemosiderin deposition (Fig. 8b) compared to normal mice (Fig. 8a). However, sections from the treated mice groups demonstrated a gradual decrease in hemosiderin deposition patches (Fig. 8c, d, e respectively). The highest treatment dose exhibited a parallel effect to the standard desirox-treated group (Fig. 8f). Accumulated collagen in liver was also stained blue using Masson’s trichrome. The microscopic observation suggested that the liver section of control mice revealed normal lobular architecture and distribution of collagen (Fig. 9a). From the liver section of iron-overloaded mice, it was evident that the normal architecture of the liver was destroyed and the nodules surrounded by accumulated collagen indicating fibrous cirrhotic (Fig. 9b). However, after treatment with SPW1, a gradual decrease in the degree of collagen deposition was observed (Fig. 9c, d, e respectively). Here also, the treatment with the highest dose demonstrated a similar scenario when compared to the standard desirox-treated group (Fig. 9f).

#### Phytochemical analysis

**Fig. 10:**
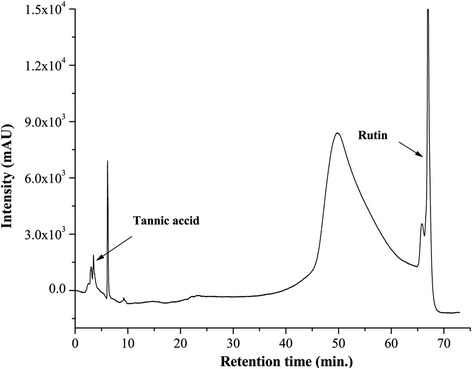
HPLC analysis of SPW1. The marked peaks denote the retention of peaks matched with the retention of the respective standard phytochemicals in the same condition

Qualitative determination of the phytochemicals ensured the presence of phenolics, flavonoids, carbohydrates, tannins, glycosides and terpenoids (Table 4). However, quantitative analysis revealed that phenolics and glycosides dominated over the other phytochemicals tested (Table 4). HPLC analysis was done to identify the probable bioactive compounds comparing the retention time of the standard compounds. Major peaks with retention time 3.5, 6.1, 50.12, 67.11 min appear on the HPLC chromatogram. Among them, two peaks were identified as tannic acid (3.5 min) and rutin (67.11 min) and other peaks were remained unidentified (Fig. 10).

**Table 4:** Phytochemical analysis of SPW1

Sample	Tests	Phytochemicals									
		Phenol	Flavonoid	Tannin	Carbohydrate	Glycoside	Alkaloid	Anthra	Sap	Terpen	Triterpen
SPW1	Qualitative	+	+	+	+	+	-	-	-	+	-
	Quantitative	44.23 ± 1.08	16.65 ± 0.26	6.36 ± 0.06	10.96 ± 0.14	31.48 ± 0.09	ND	ND	ND	ND	ND

Anthra- Anthraquinone, Sap-Saponin, Terpen-Terpenoids, Triterpen- Triterpenoids. Total phenolics (mg/100 mg extract gallic acid equivalent), Total flavonoids (mg/100 mg extract quercetin equivalent), Tannin (mg/100 mg extract catechin equivalent), Carbohydtrate (mg/100 mg extract glucose equivalent), Glycoside (mg/100 mg extract Rutin equivalent), “+” represents presence of the phytoconstituent; “-” represents absence of the phytoconstituent. “ND” represents Not Determined

## Discussion

Iron, one of the essential metals needed in our animal kingdom, acts as the most common cofactor within the oxygen handling biological machinery. However, iron in excess, induced the overproduction of various free radicals which is not always effectively normalized by the internal antioxidant defense machinery caused oxidative stress related complications [11]. As our body lacks the mechanism to transmute excess iron into any other substance, the only way to stop the detrimental effect of free or partially liganded ‘iron’ is to make sure that all of its six possible ligands are satisfied, whether by endogenous chelators or those added from the diet or as pharmaceuticals [12]. The ability of the antioxidant cum iron chelating agent to reduce the toxic effect or restore the normal physiological condition is the index of its hepato-ameliorating potential, which was noticeably attained by the treatment with SPW1 in this present study.

The antioxidant capacity of SPW1 was evaluated by the decolourization of the ABTS · ^+^ and reducing property of Fe^3+^ along with the scavenging property of DPPH, stable free radicals. The assays produced an overall picture of the antioxidative property of the fraction as the total antioxidant assay, and the reducing power is complimentary to each other. The excellent DPPH radical scavenging power of the fraction gives an initial idea about the radical scavenging property.

Different ROS such as superoxide, hydroxyl and singlet oxygen radicals induced several damages to different biomolecules. Especially, superoxide radical is known as an initiator to generate other free radicals such as hydrogen peroxide or singlet oxygen in the living system [13]. Hydroxyl radicals react with the lipid membrane and produce proxy and alkoxy radical which in turn initiate the chain reaction [14]. On the other hand RNS like nitric oxide in excess causes various inflammatory diseases and when it reacts with superoxide radical it produces more toxic peroxynitrite radical that directly damages different tissues [5, 15]. From the Fig. 2, it is clear that SPW1 acted as a free radical scavenger, which effectively reduces the toxic effect of superoxide radical that in turn neutralized the threat by different ROS and RNS. Further, the radical scavenging property was supported by the in vitro iron chelation property (Fig. 3a) of the fraction as iron plays a crucial role in the generation of the free radicals.

This excellent in vitro antioxidant, free radical scavenging and iron chelation activity of the fraction leads us to test the sample for it’s in vivo iron chelation as well as hepato-ameliorating activity in Swiss albino mice. Several previous studies suggest that iron in excess, induced hepatic damage as the liver is the main storage site for iron in our body [16]. To screen the hepatoprotective potential, intraperitoneal iron-dextran injection followed by oral treatment is a reliable method. Desirox (Generic name-Deferasirox) used as positive control because it binds to iron with high affinity in a 2:1 ratio with its tridentate ligand. A study by Cheong et al. [17] showed that Deferasirox is effective in reducing serum-ferritin and liver iron concentration level in transfusional iron overload patients. In addition, it also positively recovers hematologic and hepatic function.

The leftover iron in our body is stored in the hepatic cells as ferritin or hemosiderin [18]. Thus, measurement of the iron levels in liver is used for diagnosis the iron-load in iron overloaded disease. In contrast, ferritin, a ubiquitous intracellular iron-binding protein in serum, generally stores iron in a non-toxic ferric form and releases it in a controlled fashion whenever needed [19]. So a level of serum ferritin is an indirect key marker revealing the amount of hepatic iron content. The highest dose of SPW1 significantly eliminated the surplus iron justify its iron chelation property. On the other hand, to overcome iron overloaded state, various readily available iron chelator drugs were administered, but many of them struggle with a narrow binding capacity for ferric iron (Fe^3+^). The Fig. 3b signified that increasing concentrations of SPW1 significantly released iron (Fe^3+^) from ferritin with time and the correlation between the percentage of iron released from ferritin and the reducing power (Fig. 3c) was quite significant (R^2^ = 0.804). This result also validates that SPW1 alone effectively chelate the excess iron by reducing it from its ferric form.

Levels of serum enzyme are checked in the clinical diagnosis to determine the condition of various diseases and tissue injury [20, 21]. As these enzymes are predominantly found in the hepatic cell, liver damage due to excess iron leads to the release of these intracellular enzymes into the blood [22] as evident by increased serum parameters (Table 3). The administration of SPW1 has reduced the increased level of the serum marker enzymes almost similar to the standard desirox. This result indicated towards its healing capabilities of hepatic parenchyma and regeneration of hepatocyte as well as its functional efficiency [23].

To combat with the excess free radicals, living cells are armed with a group of intracellular antioxidant enzymes such as GST, SOD, CAT and the small compound GSH, which act as a first line of defenses. So estimation of the levels of these antioxidant enzymes is a proper indirect way to assess pro-oxidant-antioxidant combat in tissues [24]. Iron-overloaded condition stimulates the oxidative stress followed by the reduction of the levels of antioxidant enzymes. Oral treatments significantly restore the levels of the entire antioxidant enzymes to normal more readily than used drug desirox. This result signified the fraction SPW1 as a potent antioxidant agent both in vitro as well as in vivo condition.

The iron-overloaded condition increases the disease related serum enzymes and reduces the activity of cells antioxidant defense machinery, ultimately leading to liver tissue damage. The lipid bilayer is destroyed by hydroxyl radical (lipid peroxidation) and results to end products such as malondialdehyde (MDA) which in turn activate the stellate cells to initiate liver fibrosis [25–27]. After liver injury, the production of collagen predominates over hepatocellular regeneration as an immediate healing response, thereby occupying the injured areas instead of destroyed hepatocytes. Collagen content is therefore considered to be a major marker of liver fibrosis and hepatotoxicity [28]. Iron-induced liver pathogenicity also leads to the oxidation of various important structural and functional proteins and forms protein carbonyls. Thus, it serves as a marker of oxidative stress and leads to the onset/development of various diseases including cystic fibrosis and ulcerative colitis [29]. The chelation of excess iron by SPW1 subsequently reduced the liver damage and liver fibrosis indicating its hepato-ameliorating potency as shown in Fig. 6 which is again better than desirox.

Above all, liver biopsy is the ultimate standard to determine the degree of tissue damage and fibrosis alongside other pathological tests. The live sections stained with hematoxylin and eosin exhibited various degrees of inflammation, necrosis as well as cell wall degeneration in iron overloaded condition, but treatment with SPW1 reduced the tissue damage that also supported the result of the restoration of serum enzyme and antioxidant enzyme levels. Perls’ Prussian blue staining revealed the visual confirmation of iron chelation property of the test sample along with the liver iron content and serum ferritin content. On the other hand, Masson’s trichrome staining disclosed the reduction of liver fibrosis as the collagen content gradually decreased with the increasing doses of SPW1, which also confirmed the anti-fibrotic effect of the fraction along with the test for collagen content.

The presence of different phytochemicals confirmed the backbone of the bioactivity of SPW1. The adequate amounts of phenolic and glycosides in SPW1 revealed that the fractionation procedure mainly concentrated the glycosidic phenolics and flavonoids from the water fraction. This observation was also confirmed by the HPLC analysis, where only two compounds were identified, one of them is tannic acid (phenolic) and other is rutin (glycoside) and the major peak at 50.12 min is probably the combined peak of the other glycoside compounds. Literature survey revealed that, tannic acid, rutin and other glycosides are potent antioxidants, metal chelators thereby inhibiting lipid peroxidation, which also support their hepato-ameliorating potentials [30, 31]. Especially, rutin, a flavonoid glycoside, is found in wide variety of plants exhibit powerful anti-inflammatory activity along with its antioxidant, metal chelation and protective effects on hepatotoxicity [32].

## Conclusions

From the present study, it could be concluded that SPW1 can be used as a favorable candidate to cure iron overload-mediated oxidative stress and hepatotoxicity as it is capable of both scavenging the free radicals and chelate the excess iron in the body. It also further concluded that the activity of SPW1 is mainly due to the glycosidic phenolic and flavonoid compounds present in it. Though, further studies are warranted to determine the exact mechanism(s) involved in the hepatoprotective activity of the SPW1 along with the complete characterization of all bioactive compounds present in this fraction.

## Abbreviations

ALAT, alanine aminotransferase; ALP, alkaline phosphatase; ASAT, aspartate aminotransferase; b.w., body weight; BSA, bovine serum albumin; CAT, catalase; CNCI, Chittaranjan National Cancer Institute; CPCSEA, Committee for the purpose of control and supervision of experiments on animals; CRIA, Central Research Institute of Ayurveda; DPPH, 2,2-diphenyl-1-picrylhydrazyl; EDTA, Ethylenediamine tetraacetic acid; ELISA, Enzyme-Linked Immunosorbent Assay; g, Gram; GSH, reduced glutathione; GST, glutathione-S-transferase; HPLC, high performance liquid chromatography; i.p, intraperitoneal; kg, kilogram; M, molar; MDA, malondialdehyde; mg, milligram; ml, milliliter; mM, millimolar; RNS, reactive nitrogen species; ROS, reactive oxygen species; SD, standard deviation; SOD, superoxide dismutase; TBARS, thiobarbituric reactive substances
